# Distal biceps reconstruction: a long-term follow-up of the complications and durability of the single-incision power optimizing cost-effective (SPOC) repair

**DOI:** 10.1016/j.jseint.2023.07.016

**Published:** 2023-08-08

**Authors:** Jefferson Li, Lucas M. Seiler, Nathan A. Hoekzema, Toby R. Johnson, Julia Lee, Joanne L. Ridenauer, Cary M. Tanner

**Affiliations:** a University of California-Fresno, Fresno, CA, USA; b Revere Health, Provo, UT, USA; c Sierra Pacific Orthopedics, Fresno, CA, USA

**Keywords:** SPOC, Distal bicep, Repair, Reconstruction, Complications, Strength measurement

## Abstract

**Background:**

The Single-Incision Power Optimizing Cost-Effective Repair (SPOC) method reattaches the distal biceps tendon to its original posterior anatomic footprint and utilizes the anterior cortex of the supinated radius for fixation. The purpose of the study was to define the long-term complications and durability of the SPOC method.

**Methods:**

Two hundred and eighteen patients underwent the SPOC repair of distal biceps ruptures from 2008 to 2020, with 185 having at least 1-year follow-up data. The average follow-up was 50.1 months. Information regarding smoking, body mass index, interval between injury and surgery, peripheral nerve injury, heterotopic ossification, vascular injury, re-rupture, chronic regional pain syndrome, fracture of the radius, loss of motion, pain with use, and deformity were acquired.

**Results:**

No complication occurred beyond the third postoperative month. No patient complained of severe lateral antebrachial cutaneous nerve-related symptoms. Major complications exclusive of re-rupture occurred include 1 case of heterotopic ossification and 1 deep infection. Major complications with re-ruptures occurred in 9 patients (4.8%). Seven of the re-ruptures (78%) were associated with an unexpected forceful contraction within the first 4 weeks postop. All complications aside from 1 minor complication occurred in the chronic group. Long term follow-up revealed no re-ruptures and high satisfaction rate with return of strength, motion, and biceps profile.

**Conclusion:**

The safety profile of the SPOC repair is consistent with those of other published repairs. Major complications were associated with prolonged intervals between injury and reconstruction. Re-ruptures were associated with worker’s compensation status and patient noncompliance with postoperative protocols.

The Single-Incision Power Optimizing Cost-Effective Repair (SPOC) described in 2013 restores supination strength, endurance, and power following a distal biceps reattachment.^18^ The technique reattaches the distal biceps tendon to its native footprint, thus maximizing its moment arm and preserving the radial tuberosity’s cam effect (Fig. 1*A*).^14^, ^15^, ^16^

**Figure 1:**
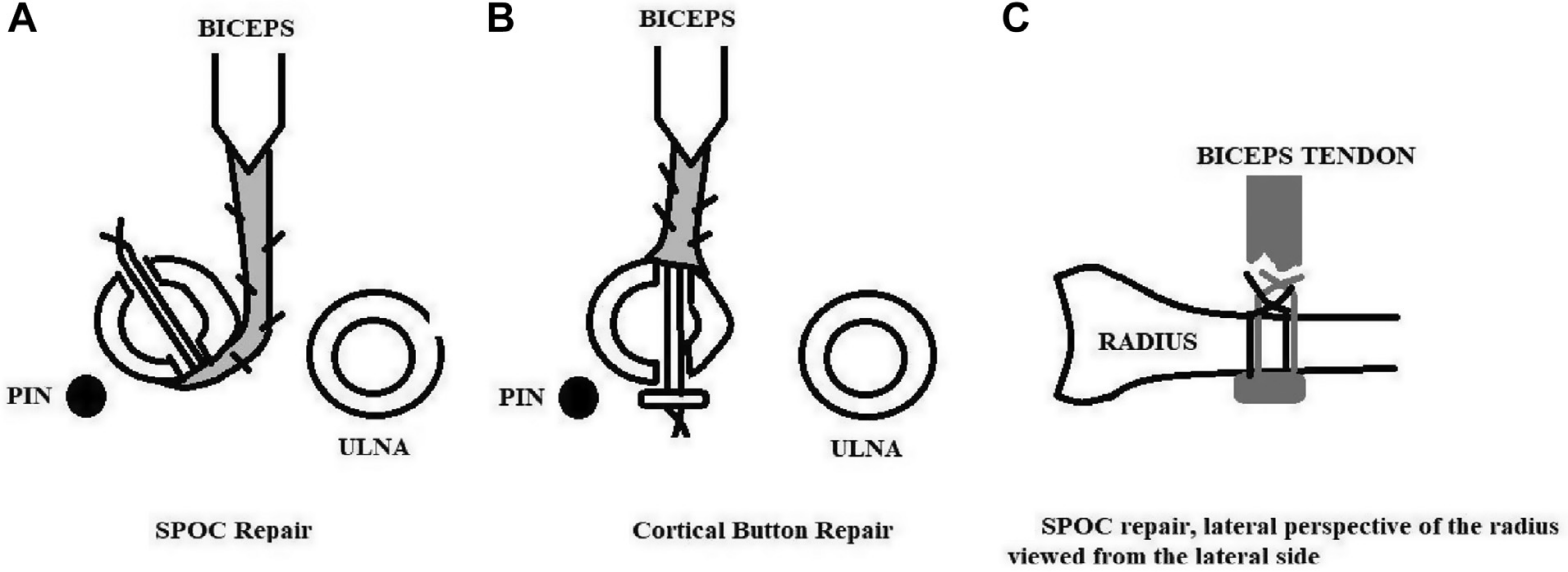
A: SPOC repair. B: Cortical button repair. The radius is in supination in A and B: the tuberosity would be prominent on an anterior-posterior (AP) radiograph. A: The SPOC technique reverses the cortical button tendon insertion and fixation site, so replacing the tendon to its native insertion site and providing fixation to the anterior cortex of the radius. This restores the supination moment arm. In addition, the proximal radial to distal ulnar drill trajectory increases the distance from the posterior drill hole to the posterior interosseous nerve (Fig. 2).^12^^,^^20^ C: The SPOC method incorporates 2 separate sutures securing the distal biceps tendon to the anterior cortex of the radius, each tied over the bone bridge. *SPOC*, Single-incision power optimizing cost-effective repair.

Schmidt has stated that “future directions for distal biceps tendon repair techniques should focus on restoring the anatomic reattachment site while limiting supinator damage”.^14^ Tadevich noted that restoration of the functional strength following a distal biceps tendon rupture is a “direct function of the attachment position relative to the anatomic footprints”.^17^

Traditional single-incision button or anchor repairs of the distal biceps tendon are a tendon transfer to the visible anterior cortex of the radius when the forearm is fully supinated (Fig. 1B). This repair method can lead to the supination weakness because it forgoes the cam effect of the radial tuberosity.^14^, ^15^, ^16^ The SPOC method secures the torn distal biceps tendon to its native insertion by using an anterior bone bridge for fixation (Fig. 1 *A* and *C*).^18^ Biodex testing demonstrated that it is capable of restoring more than 90% of the peak supination strength to the endpoint of supination, supination endurance, and supination power without loss of motion.^18^ A full arc of supination strength, endurance and power is needed to successfully loosen and tighten fasteners, control motorized drills, swing bats, and golf clubs (Table I). The SPOC technique can also be used in distal biceps reconstructions to attach the Achilles allograft to the radius. In our experience, this method of Achilles tendon distal biceps reconstruction often results in near full recovery of supination strength throughout a full forearm range of motion.^12^

**Table I:** Requirements of a successful distal biceps repair in those patients using rotational tools.

Biomechanical parameter	Example of relevant work task
Peak supination strength	a) Loosening or tightening fasteners b) Controlling rotation of motorized drill, particularly when using large diameter bits that bite into the material, thus transferring rotational force to the patient’s wrist upon sudden stop of the drill bit
Supination endurance	a) Correlates with total degrees of rotation that a rotational task requires: Installing a bolt, tightening a bolt, finishing drilling task
Supination power	a) Correlates with the time it takes to complete a task
Recovery of range of motion	a) Maximizes the rotation of a tool with each muscle contracture

Four studies comprising more than 5500 distal biceps tendon patients have an overall complication rate in the range of 25%, with major complications occurring approximately 7% of the time. Major complications exclusive of tendon re-rupture occurred, on average, in 5% of patients.^1^^,^^2^^,^^4^^,^^20^ As noted by Ford, it is helpful to separate the complications into those that are unlikely to be of significant consequence to the patient from those that are significant (Table II).^4^ Long-term outcomes for the SPOC repair have not been elucidated. The purpose of this study was to assess patient’s satisfaction with their biceps repair. Questions about strength, biceps profile, and pain with use were asked primarily to reveal symptoms that would suggest repair failure.

**Table II:** A modified Ford stratification of the complications of distal biceps repair procedures by clinical severity.

Impact to patient	Complications
Minimal patient impact	a) Mild to moderate numbness in the LABC Distribution b) Heterotopic ossification that does not limit motion c) Superficial infection d) Hematoma that does not require intervention
Severe patient impact	a) Heterotopic ossification that impairs motion b) Major peripheral nerve injury including severe LABC symptoms c) Chronic regional pain syndrome d) Vascular injury e) Deep infection f) Hematoma requiring decompression g) Fracture of the radius h) Compartment syndrome i) Chronic regional pain syndrome
Patient influenced complications	a) Re-rupture of the repair: A majority of the reported re-ruptures have been related to patient compliance

*LABC*, lateral antebrachial cutaneous nerve.

## Materials and methods

This study is a retrospective review of 185 SPOC repairs of distal bicep tendon ruptures. All distal biceps tendon repairs from 2008 to 2020 were retrieved by CPT code 23432. Their charts were screened for repair method and duration of follow-up. All of the charts were reviewed, and the patients were called or examined in person. Exclusion criteria include those with less than 6-month follow-up. Two hundred and fifteen patients were identified. Thirty-three patients had less than 6 months follow-up, leaving 185 patients to serve as the basis for this study.

Three board-certified hand surgeons (T.R.J, J.L, C.M.T) performed all of the 218 SPOC repairs^18^ from 2008 to 2020. These authors diagnosed the distal biceps injury based on patient history, physical examination, and review of the preoperative magnetic resonance imaging (MRI) obtained in all cases. Single-Incision Power Optimizing Cost-Effective Repairs were used for native distal biceps repairs and Achilles tendon allograft reconstructions. The indications for Achilles allograft use were chronic partial or complete tears that propagated proximally into the musculotendinous junction, leaving little to no healthy tendon for suture fixation, and for chronic tears requiring greater than 100 degrees of elbow flexion for reattachment after débridement of unhealthy tendon. The original technique was described in 2013.^18^ Briefly, a standard Henry approach to the biceps tuberosity was utilized. Two 2.5-mm drill holes approximately 1 cm apart were made from the anterior radius in line with the radial tuberosity to the posterior ulnar aspect of the radius. These drill trajectories increase the distance between the drill exit and the posterior interosseous nerve as shown in the video (Fig. 2).^11^^,^^19^ #2-Nylon shuttle sutures are passed through each drill hole using an 18-gauge spinal needle. The nylon suture is retrieved posteriorly with a right angle clamp and brought out of the field anteriorly. The retracted biceps tendon is retrieved, prepared, and is secured using 2 loop whipstitches (Suturetape, Arthrex, Naples, FL, USA) such that all 4 strands exit the distal end of the tendon. Each nylon suture shuttles the suture tape ends through the drill holes in a posterior to anterior fashion. With the forearm in full supination, 1 pair of Suturetape ends are retracted by an assistant with steady traction applied, pulling the biceps tendon onto its native biceps footprint. The surgeon ties the other set of suture tape ends over the bone bridge. Once the first pair of Suturetape ends are tied, the free ends of that suture are retracted by the assistant creating additional traction. The surgeon then ties the second set of Suturetape ends over the bone bridge. Thus, 2 separate Suturetapes independently hold the biceps tendon reduced.

The postoperative protocol incorporated a fiberglass removable posterior splint to be used as comfort dictated in the first 3 weeks after surgery, but no limits of range of motion were imposed. Motion against resistance was started on the 12th postop week.

Information was obtained from the chart and patient about complications encountered with the SPOC procedure itself, the need for any additional surgery, the use of an allograft, workman’s compensation insurance, the time from injury to surgery and other complications that may have become evident. These other complications included vascular injury, neurologic injury, heterotopic ossification, chronic regional pain syndrome, loss of motion, subjective weakness, pain with use, deformity that might indicate re-rupture and fracture of the radius. Also noted was a history of smoking, the patient’s body mass index, and the partial or complete tear status of the tendon at time of surgery.

Telephone follow-up was designed to identify any complication that might have occurred beyond the patient’s final clinical visit or that degraded the initial clinical result. The primary goal of these calls was to identify any significant complications that occurred after the patients’ last clinical follow-up. Telephone calls were made by the senior author (C.M.T.) All patients were asked about their subjective strength compared to the contralateral side, baseline pain, pain with use, biceps profile compared to the contralateral side, any suspicion of re-rupture, any further surgery related to their biceps tendon, and their satisfaction with the procedure.

Fisher’s exact test was utilized to assess the prevalence of obesity and smokers between patients who did and who did not have postoperative complications. Fisher’s exact test was also used to compare the rate of distal biceps tendon re-rupture within Workman’s Compensation patients and that of the rest of our study population. The study was performed with Institutional Board Review: Community Medical Centers Institutional Review Board; IRB #2011025.

## Results

The average in-person follow-up was 20.6 months, with telephone follow-up averaging 75.6 months postop. Overall average follow-up was 50.1 months. There were 5 female patients. Seventy-four patients (40%) were cared for under the workman’s compensation system. The average time from the original injury to the repair was 4.53 months (2 weeks to 24 months) with a median interval of 2.5 months. The average time from injury to surgery for those requiring an Achilles allograft was similar: 4.1 months with a median of 3 months.

The incidence of tobacco use and body mass index in the overweight to obese range were similar between patients that sustained a complication compared to those that did not (Table III). The rate of complication profiles between native tendon repairs compared to Achilles allograft augmentation was similar. Table IV is the summary of complications. Complications are presented in 3 groups: the entire cohort, the time between injury and surgery, and those requiring Achilles tendon allografts.

**Table III:** The incidence of tobacco use and obesity in patients with and without a complication.

All patients	Patients with complications	*P* value
94% obese/overweight	100% obese/overweight	1.0
11% smokers	10% smokers	.71

**Table IV:** Complications: percentages expressed as percentage of subgroups.

A) Total study population, 185 patients		
	Complication, % of patients	Complication, n of patients
Overall complications	21.1%	39
Minor complications overall	15.2%	28
LABC numbness minimal	10.8%	20
LABC numbness moderate	2.2%	4
Superficial hematoma	1.1%	2
Keloid	1.1%	2
Minor heterotopic ossification	0.5%	1
Major complications overall	5.9%	11
Biceps tendon re-rupture	4.8%	9
Exclusive of tendon re-rupture	1.1%	2

B) Acute / Subacute repairs (time to surgery < 3 weeks), 54 patients		
	Complication, % of patients	Complication, n of patients
Minor complications	2%	1
Minor heterotopic ossification	2%	1
Major complications	0%	0
	0%	0

C) Chronic repairs (time to surgery > 3 weeks), 131 patients		
	Complication, % of patients	Complication, n of patients
Overall complications	29.7%	39
Minor complications overall	21.3%	28
LABC numbness minimal	15.3%	20
LABC numbness moderate	3.1%	4
Superficial hematoma	1.5%	2
Keloid	1.5%	2
Minor heterotopic ossification	0%	0
Major complications overall	8.4%	11
Biceps tendon re-rupture	6.9%	9
Exclusive of tendon re-rupture	1.4%	2

D) Chronic repairs with Achilles tendon allograft, 43 patients		
	Complication, % of patients	Complication, n of patients
Overall complications	23.3%	10
Minor complications overall	16.3%	7
LABC numbness minimal	9.3%	4
LABC numbness moderate	2.4%	1
Superficial hematoma	2.4%	1
Keloid	2.4%	1
Minor heterotopic ossification	0%	0
Major complications overall	7.0%	3
Biceps tendon re-rupture	7.0%	3
Exclusive of tendon re-rupture	0%	0

E) Chronic repairs without Achilles tendon allograft, 88 patients		
	Complication, % of patients	Complication, n of patients
Overall complications	33%	29
Minor complications overall	24%	21
LABC numbness minimal	18%	16
LABC numbness moderate	3.4%	3
Superficial hematoma	1.1%	1
Keloid	1.1%	1
Minor heterotopic ossification	0%	0
Major complications overall	9.1%	8
Biceps tendon re-rupture	6.8%	6
Exclusive of tendon re-rupture	2.3%	2

*LABC*, lateral antebrachial cutaneous nerve.

No complications were identified after the third postoperative month. Telephone follow-up did not identify any additional biceps tendon re-ruptures. No patients reported undergoing further surgeries related to the biceps tendon. Subjectively, 139 patients (75%) reported full strength in their operative arm. Forty-four patients (24%) reported recovery of functional strength, but less than their preinjury state. No patient had pain that limited use. Cosmetically, 144 patients (78%) felt that their biceps profile was restored compared to the contralateral side. Thirty-one patients (17%) reported that a mild deformity existed. Ninety-four percent of patients reported that they would have the procedure again and would recommend it to a friend with a distal biceps rupture.

### Overall major complications (5.9% of study population)

There were 2 patients (1.1%) with major complications exclusive of re-ruptures. One patient (.5%) developed heterotopic ossification that limited motion. Two resections of the heterotopic ossification were performed at 18 months and 36 months after the index procedure. Full flexion, extension and pronation were obtained following the second resection. Supination improved from minimal supination to 25 degrees shy of full supination compared to the contralateral side. The other patient (.5%) developed a deep methicillin resistant staphylococcus aureus infection that required removal of all of the suture material to control the infection. The patient elected not to undergo a revision.

Of the 9 re-ruptures (4.8%), 7 patients (78%) were able to define a forceful contraction that resulted in the re-rupture that was not in compliance with the postop protocol within the first 4 weeks of surgery. Seven of the 9 patients (78%) with re-ruptures were cared for under the workman’s compensation system. Workman’s compensation patients had a higher rate of re-ruptures compared to the rest of our patient population (*P* = .04). One patient chose not to have a revision but 8 were revised with 88% achieving excellent results. There were no other non-lateral antebrachial cutaneous nerve (LABC) neurologic injuries, no cases of CRPS, no vascular injuries and no case of radial fracture through the drill holes. The incidence of major complications did not appear to be influenced by the degree of distal biceps tear at surgery: 5 major complications occurred with the repair of partial tendon tears and 6 with complete tears.

### Overall minor complications (15.2% of study population)

Twenty patients (10.8%) reported mild LABC numbness and 4 patients reported moderate LABC numbness (2.1%). None of these symptoms caused a functional challenge for any patient, including the 1 patient with a known LABCN laceration. There were 2 superficial hematomas (1.1%), 2 keloid scars (1.1%), and 1 case of mild heterotopic ossification (.5%) that did not limit motion. LABC numbness appears to trend with the repair of complete as opposed to partial tears as 73% of the patients experiencing postop numbness underwent the repair of a complete tear (*P* = .14).

### Overall complications in acute repairs (injury to surgery <3 weeks; 2% of study population)

Fifty-four patients (29% of the entire group) were repaired within 3 weeks of injury. There were no major complications in tendons repaired acutely or subacutely. One patient (2%) sustained a minor complication and developed mild heterotopic ossification that did not limit motion.

### Overall complications in chronic repairs (injury to surgery > 3 weeks; 29.7% of study population)

In contrast to the acute repairs, these 131 patients (71% of the entire group) experienced the vast majority of the complications. Complications occurred in 39 (29.7%) patients with chronic repairs. Of these 39 patients, minor complications occurred in 28 (19.3%) patients and major complications occurred in 11 (8.3%) patients.

### Overall complication rate in repairs with Achilles tendon allografts (23.3% of study population)

Forty-three patients (33% of the chronic group) required the use of an Achilles tendon allograft in the reconstruction, and all were performed chronically. The complication rates of the 43 patients in the Achilles group closely reflected the complication rates in the 88 chronic patients that did not require an allograft (Table IV). Thus, it does not appear that a surgical reconstruction with an Achilles tendon allograft posed a greater complication risk than chronicity alone.

## Discussion

The SPOC repair was developed to restore the distal biceps tendon to its natural attachment site. The data presented demonstrate that patients are satisfied with their result and the majority recover full subjective strength. The total complication rate of 21.1% in this series, with major complication rate of 5.9%, compares favorably with multiple studies generally showing an average overall complication rate of 25% and major complication rate of 7%.

The proof of concept borrows from Schmidt^16^ who demonstrated that the calculated moment arm through which distal biceps tendon normally acts is on average greatest in neutral rotation (10 mm), 20% less in full pronation (8 mm), and 40% less in full supination (6 mm). Haugsvedt’s data in cadaveric forearms demonstrated a similar trend. Schmidt went on to show that the moment arm declines as the forearm rotates into supination and is markedly accentuated when anterior reinsertion sites for the reattachment of the distal biceps tendon are chosen.^7^ When an anterior insertion site is chosen, the moment arm in full pronation does not change, but it is 20% less in neutral rotation (8 mm), and falls to zero millimeters by 60 degrees of supination.^16^ Thus, when anterior repairs of the distal biceps are utilized, the biceps progressively loses its ability to supinate the forearm beyond neutral into supination.^14^ Biodex testing of the SPOC repair has demonstrated very little decline in supination torque from full pronation through full supination.^18^

Table I lists activities and tasks that require active supination. Achieving maximum supination power requires maintaining forearm supination torque through the full arc of rotation. Supination power relates to the total torque required for the task and the time it takes to complete it. Some tasks such as controlling rotational drills require isometric supination strength at a variety of forearm rotational positions. These tasks are optimized with anatomic biceps tendon repairs as the strength from neutral to supination is maintained.

Kelly et al^10^ defined acute repairs as those done within 10 days of injury, subacute if done within 3 weeks of injury and chronically thereafter. A delay between injury and surgery was shown to be associated with increased complication rates, but stratifying complication rates according to the time from injury to surgery has not always been reported. This study revealed similar findings. In the 54 (29%) patients repaired within 3 weeks of injury, there was 1 minor complication and no major complications.

Two incision approaches also replace the distal biceps tendon to it anatomic footprint. A previous clinical study compared the difference between anterior button-style repairs and extensor carpi ulnaris splitting, 2-incision repairs.^14^ There was significantly greater supination strength at 60 degrees of supination in the 2 incision repairs which is consistent with the biomechanical data indicating the moment arm decline in anterior repairs.^14^ It has always been our assumption that either a 2-incision method or the SPOC method would achieve the same supination peak strength, supination endurance, and power results. However, 2 factors regarding the performance of the 2-incision method have emerged beyond the historic concern over the development of heterotopic ossification. Traditional 2-incision techniques have used a trough in the region of the radial tuberosity into which the distal biceps tendon is inserted. Avoiding such a trough improves the biceps moment arm and has been shown to increase supination strength.^15^ The 2-incision technique has also been called further into question as the method splits the supinator. There have been reports of visible atrophic changes within the supinator muscle noted on postoperative MRI’s.^14^

The SPOC method restores the normal biceps footprint through a single anterior incision without disturbing the posterior musculature. More work is likely needed to sort out the clinical importance of the MRI changes noted in the supinator with 2-incision methods. It should be noted that 2 groups have published modifications of the SPOC method: 1 using an arthroscope and another using a specialized tunneling device to facilitate the repair.^6^^,^^13^

Re-rupture or loss of fixation represents a unique major complication. Most re-ruptures have been shown to occur very early in the postoperative period and are typically associated with an unexpected forceful contracture of the biceps.^3^^,^^5^^,^^8^^,^^9^ These contractures technically constitute noncompliance, but in our study many were non-volitional. Re-ruptures are typically reversible with revision repair. Other major complications may not enjoy the same ease of reconstruction and may confer permanent loss of function. In this study, re-ruptures were associated with workman’s compensation status. However, the overall number of patients with re-ruptures was small, so the significance of this finding is unclear.

There are a few limitations of this study. The first is that it is a retrospective review without a control group. Other limitations include generalizability, as this study was done at a single center that originated the technique. We are encouraged by the results reported by Tadevich^17^ that are consistent with the strength and safety profile detailed above. The subjective weakness in our patients was not quantified by a standardized measurement. Finally, it is possible that there were patients with undetected partial tears as MRIs were not routinely obtained on postoperative patients.

## Conclusion

The SPOC method restores the anatomic footprint of the distal biceps tendon without disturbing the supinator muscle. The majority of patients reported a high satisfaction rate with subjective strength, motion, and restoration of the biceps profile. Our study’s long-term follow-up demonstrates complication rates consistent with other repairs in the literature. All complications in our cohort occurred within the first 3 months. Acute repairs had fewer complications than chronic repairs. All of the complications except 1 minor complication occurred in repairs performed chronically (> 3 weeks) from the date of surgery. The use of Achilles allograft, patient’s obesity, and smoking did not increase the risk of complications.

Complications following the repair of the distal biceps appear to occur early in the postoperative course. Re-ruptures were associated with workman compensation coverage and forceful muscle contractures of the repair early within the first month of recovery. Future studies may reveal that it is possible to reduce the number of re-ruptures through patient education and modified rehabilitation protocols.

## Disclaimers

Funding: No funding was disclosed by the authors.

Conflicts of interest: The authors, their immediate families, and any research foundation with which they are affiliated have not received any financial payments or other benefits from any commercial entity related to the subject of this article.

## Supplementary Data

Figure 2 The video demonstrates how the SPOC method results in the anatomic reinsertion of the biceps tendon into its native posterior footprint from a single anterior incision. In addition, the utility of the proximal radial to ulnar distal drill trajectory so increasing the distance to the PIN is shown.
